# Commentary: Production and Characterization of Monoclonal Antibodies to Human Interleukin 2: Strategy and Tactics

**DOI:** 10.3389/fimmu.2015.00454

**Published:** 2015-09-02

**Authors:** Kendall A. Smith

**Affiliations:** ^1^Division of Immunology, Department of Medicine, Weill Medical College, Cornell University, New York, NY, USA

**Keywords:** T cell growth factor (TCGF), interleukin-2 (IL-2), T cell clones, IL-2 receptor

Although we had successfully created antigen-specific cytolytic T lymphocyte lines (CTLLs) using conditioned media as a source of growth-promoting factors, we had no effective method to determine the relative activities of different batches of conditioned media. Therefore, it was crucial to create a quantitative assay for the activity we termed “T cell growth factor” (TCGF). Fortunately, Torgny Fredrickson and I had already created a bioassay for the red blood cell growth factor, erythropoietin (EPO), using murine fetal liver cells that are enriched for EPO-responsive precursors (1, 2). Thus, patterned on the EPO bioassay, it was straightforward to construct a similar assay for TCGF using as target cells our long-term CTLL. The critical elements of the assay were (1) a low density of CTLL target cells and (2) serial twofold dilutions of conditioned media samples, thereby establishing a dose–response curve that allowed comparison of separate conditioned media (3). Of note was the observation that the curve was symmetrically sigmoid when the linear responses of tritiated thymidine incorporation were plotted vs. the logarithm of the conditioned media dilutions. We arbitrarily assigned 1.0 U/mL that yielded 50% of maximal growth promotion at a dilution of 1:10. This assay represented the first ever quantitative bioassay for a lymphokine.

Thus, armed with a rapid, quantitative bioassay, we next sought to generate T cell clones derived from our antigen-specific CTLL so that we could assess the potential problem of target cell heterogeneity. We tried two established cloning methods: (1) dilute cell suspensions seeded into soft agar containing TCGF-conditioned media and (2) limiting dilution (0.03–0.01 cells/well) in microtiter plates containing TCGF-conditioned media. The limiting dilution technique in suspension culture worked very well, yielding 67–100% plating efficiency. This was the first description of monoclonal antigen-specific cytolytic T cells (4). Accordingly, T cell clones permitted an unambiguous interpretation that TCGF was acting directly on cloned T cells and not indirectly through an intermediate cell type, e.g., an APC. We submitted our manuscript to *Nature*, which again rejected it without review [see Ref. (5)], so that we immediately reformatted it and sent it to the *J. Exp. Med*., which accepted it without changes, so much for non-scientist journalists (Nature) vs. peer scientists (JEM) making informed editorial decisions (6). Other investigators rapidly adopted these cloning methods, in that they not only allowed for separation of the cell clones, but also could be used to grow large numbers of progeny, which could be used for both biological and molecular characterizations. The ability to create monoclonal functional T cells was as transformative for T cells as monoclonal antibodies were for B cells.

The foregoing results of these studies on the various mitogenic activities in conditioned media pointed to the overwhelming need to identify the molecules responsible for the bioactivities. In addition, the molecular mechanisms whereby the mitogenic activities interacted with their target cells loomed as a huge overriding question. Thus, armed with the quantitative TCGF bioassay, analytical biochemical experimental approaches yielded results consistent with a single, small protein (~15.5 kDa; pI = 8.2) as solely promoting the biological response (7). This was an important finding, in that it meant that further purification of the molecule responsible for the activity would be straightforward, whereas if several molecules cooperated to produce the activity, purification of each component would be difficult.

These biochemical approaches permitted the separation and purification of enough biosynthetically radiolabeled TCGF to permit classic hormone binding assays, which revealed that radiolabeled TCGF-binding sites expressed all of the characteristics of true hormone receptors, i.e., the binding was restricted to TCGF-responsive cells, there was a lack of competition by other growth factors and hormones, the binding was of very high affinity, and there was a close correlation between the TCGF concentrations that bound to cells and those that mediated the proliferative response (8). These data all supported the conclusion that the binding site detected was on the receptor through which the biological effects of TCGF are initiated. This report was the first to demonstrate and characterize a cytokine receptor, and consequentially became the prototype for the identification and characterization of all subsequent cytokine receptors involved in regulating immune and inflammatory responses. Moreover, the radiolabeled TCGF binding assay was absolutely instrumental in the identification of the first monoclonal antibody reactive with a cytokine receptor molecule (9).

These experimental approaches allowed us to calculate the Specific Activity of IL-2 for the first time, so that 1.0 U/mL = 150 ng/mL, which indicated that we would need to start with several liters of conditioned media to concentrate and purify adequate IL-2 protein to immunize mice with microgram amounts of TCGF protein and ultimately to develop the first monoclonal antibodies reactive with a lymphokine molecule (10). Prior to the development of quantitative bioassays and radioreceptor assays, investigators had tested only one dilution of a sample, e.g., a 1:2 or a 1:4, so that they could not quantify the amount of activity and relate it to a measured protein concentration. Thus, attempts were made to purify cytokine molecules with only 10–100 mL of starting conditioned media. In this article, we detailed the critical experimental approaches and advances that led to our success in generating monoclonal antibodies reactive with human TCGF, so that others might follow. Through rigorous biochemical analytic methods, it was possible to prove that the monoclonal antibodies were useful to immunoaffinity purify IL-2 molecules to homogeneity in milligram quantities from multiple liters of conditioned media that then could be used for unambiguous molecular and biological characteristics of the first interleukin molecule to be identified.

Thus, with these new and novel cellular and molecular reagents, we could proceed to experiments that had never been done before.

## Conflict of Interest Statement

The author declares that the research was conducted in the absence of any commercial or financial relationships that could be construed as a potential conflict of interest.
